# Social Media Activities With Different Content Characteristics and Adolescent Mental Health: Cross-Sectional Survey Study

**DOI:** 10.2196/73098

**Published:** 2025-04-28

**Authors:** Chia-chen Yang, Paul Hunhoff, Yen Lee, Jonah Abrell

**Affiliations:** 1 School of Educational Foundations, Leadership and Aviation Oklahoma State University Stillwater, OK United States; 2 Department of Health Professions Education Uniformed Services University of the Health Sciences Bethesda, MD United States; 3 School of Community Health Sciences, Counseling and Counseling Psychology Oklahoma State University Stillwater, OK United States

**Keywords:** social media, depression, anxiety, social support, approval anxiety, social comparison, adolescence

## Abstract

**Background:**

Adolescent mental health concerns are rising in the United States, with social media often cited as a contributing factor, although research findings remain mixed. A key limitation is the simplistic view of social media use, which fails to consistently predict well-being. Scholars call for a more nuanced framework and a better understanding of how social media use influences adolescent mental health through various psychosocial mechanisms.

**Objective:**

Using the Multidimensional Model of Social Media Use, we explored how 4 activities with various content characteristics (intimate directed communication, intimate broadcasting, positive broadcasting, and positive content consumption) are associated with depression and anxiety through 3 psychosocial mediators: social support, approval anxiety, and social comparison.

**Methods:**

Cross-sectional survey data were collected through Qualtrics’ panel service from a sample of adolescents whose gender and racial or ethnic distributions were nationally representative (N=2105; mean age 15.39, SD 1.82 years). Participants passed attention checks to ensure data validity. Measures included 9 validated scales (Cronbach α=0.83-0.91): 4 social media activities (intimate directed communication, intimate broadcasting, positive broadcasting, and positive content consumption), 3 mediators (social support, approval anxiety, and social comparison), and 2 mental health outcomes (depression and anxiety). Using Mplus, we performed 2-step structural equation modeling. Confirmatory factor analysis established scale validity, and path analysis tested the hypothesized and exploratory associations between social media activities, mediators, and mental health, controlling for demographic covariates and the amount of phone use. Model fit criteria (comparative fit index and Tucker-Lewis index were close to or greater than 0.95; root mean square error of approximation was less than 0.08) were met. Significance was determined using a false discovery rate control, with the familywise type 1 error rate set at 0.05.

**Results:**

Our findings showed that positive broadcasting was associated with lower depression (β=–.14; *P*<.001) and anxiety (β=–.06; *P*=.03), mainly through the direct paths. The other 3 activities were related to more mental health problems. Specifically, intimate directed communication was associated with greater depression (β=.06; *P*=.03) and anxiety (β=.06; *P*=.04); intimate broadcasting was associated with greater anxiety (β=.07; *P*=.02); and positive content consumption was related to higher depression (β=.13; *P*<.001). Approval anxiety or social comparison played a salient role in these total effects.

**Conclusions:**

The findings highlight the importance of distinguishing social media activities when assessing risks and benefits. Intimate directed communication, intimate broadcasting, and positive content consumption became risk factors for increased anxiety and depression through approval anxiety, social comparison, or both. Positive broadcasting was related to better mental health because of its direct associations with lower depression and anxiety.

## Introduction

### Background

US adolescents are facing increasing mental health challenges [1], and social media has been proposed as a contributor to this trend. However, the findings on the relationship between social media use and adolescent mental health have been mixed and inconclusive [2,3]. Despite a wealth of research in this area, the existing literature has 2 noticeable limitations. First, the dominant conceptualization of social media use has been overly simplistic, which hampers the reliability and interpretability of findings. Initially, researchers focused on general aspects of social media use, such as its amount, frequency, or intensity [4]. More recently, attention has shifted to distinguishing between active and passive use (ie, creating vs consuming content), with arguments suggesting that active use supports mental health while passive use detracts from it [5]. Nonetheless, neither general use nor this active-passive distinction consistently predicts users’ well-being [3,6], indicating a need for a more refined conceptual framework. Scholars have called for more nuanced activity categories and greater attention to the content of these activities [3,7,8]. Second, there has been a lack of studies that concurrently examine multiple protective and risk mechanisms, which limits our understanding of how social media use interacts with various psychosocial factors to influence adolescent mental health.

To address these gaps and respond to recent scholarly calls, we drew on the Multidimensional Model of Social Media Use (MMSMU) [9] to conceptualize social media use and identify major mechanisms. We distinguished 4 specific social media activities (intimate directed communication, intimate broadcasting, positive broadcasting, and positive content consumption) and investigated 3 psychosocial processes that have been theorized to either promote or undermine users’ mental health (social support, approval anxiety, and social comparison). By examining these specific social media activities through both protective and risk mechanisms, this study offers a more comprehensive understanding of the complex relationship between social media and adolescent mental health, ultimately contributing to a more informed basis for future research and intervention development.

In this study, social media is defined broadly to encompass applications, websites, and devices that facilitate different forms of individual and group interactions, including but not limited to messaging, texting, and social networking sites. We focused on depression and anxiety as indicators of mental health given they are among the primary contributors to adolescent illness and disability [10].

### Conceptualizing Social Media Activities

The MMSMU [9] highlights the necessity of exploring how various facets of social media use impact users’ well-being. It categorizes social media activities into 3 distinct types: directed communication, broadcasting, and content consumption. Directed communication focuses on interactions with specific individuals, characterized by a 2-way exchange, such as texting and messaging. Broadcasting targets a broader audience through posts and shares, involving active participation in generating or distributing content. Finally, content consumption is the act of browsing and reviewing social media material, often seen as passive since it does not involve content creation or dissemination. This framework distinguishes itself from the conventional active-passive dichotomy, which lumps both directed communication and broadcasting together as active forms of social media use [5]. By acknowledging the differences between these two activities, along with considering content consumption, the MMSMU facilitates the exploration of more nuanced social media dynamics.

While the MMSMU [9] touches on content characteristics of various social media activities, these characteristics are not a formalized component in the model. Recognizing how content characteristics can shape the nature of these activities and thus their implications, we refined the conceptualization of social media activities by specifying the focal content characteristics of interest. This approach should enhance the conceptual clarity of our study.

For directed communication, we focused on intimate interaction. Intimate online practice refers to revealing personal, private, and sensitive self-information, with some having the potential to make oneself vulnerable [11]. This includes openly sharing one’s feelings and emotions, thoughts, personal issues or experiences, and even weaknesses. Intimate self-disclosure plays a crucial role in relationship development and support acquisition [12,13]. Much research has shown that directed communication facilitates well-being [14-16], and it would be important to clarify whether the benefits are derived from the intimate nature.

We studied 2 types of broadcasting: intimate and positive. Positive broadcasting is the norm on social media [17,18], whereas intimate broadcasting is relatively rare [19]. With the social media affordances of asynchronicity and editability, teenagers often take the time to carefully curate their online personas, highlighting positive aspects of their lives [17,18]. The prevalence of the exercise makes positive broadcasting a relevant focus of the study. Intimate broadcasting, while less common, has particularly intriguing associations with well-being. It can both promote and dampen well-being, depending on the psychosocial processes activated [9]. It is thus important to simultaneously consider competing processes and clarify the activity’s overall impact on mental health.

Regarding content consumption, we focused on the positivity of the information encountered on social media. Given that teenagers often feel pressured to present a polished image online [18,20], it is no surprise that social media platforms are increasingly filled with a continuous stream of positive posts. This prevalence of positive content consumption makes it one of the most significant digital experiences for youth. By studying positive content consumption alongside other social media activities, we would be able to present this activity’s unique contribution to adolescent mental health. In sum, the study concerned 4 activities: intimate directed communication, intimate broadcasting, positive broadcasting, and positive content consumption.

### Social Media Activities and Adolescent Mental Health

The MMSMU [9] outlines how directed communication, broadcasting, and content consumption each have distinct implications for well-being through various psychosocial mechanisms. Within this framework, 3 mediators are particularly pertinent to the 4 social media activities examined in this study: social support, approval anxiety, and social comparison. Directed communication is thought to enhance mental health by fostering social support, while content consumption is believed to negatively impact mental health due to social comparison. Broadcasting is theorized to promote mental health through social support, although it also undermines well-being via approval anxiety. Now that we have expanded the model by identifying 4 social media activities, it is imperative to consider how they relate to mental health through these 3 prominent psychosocial processes. We review the relevant literature below.

Directed communication through social media, such as messaging and texting, is associated with various aspects of well-being, including higher self-esteem, greater moods, and diminished stress [15,16]. Social support, or the assistance and comfort received from one’s social networks [21], has been identified as a major mediator—it explains how directed communication predicts fewer depressive symptoms [14]. While most of these studies do not specify the characteristics of directed communication, similar patterns should emerge when the exercise is intimate in nature. For instance, when adolescents experience stress, if they are allowed to text their friends, the practice effectively reduces stress [16]. It is plausible that the content of the texts is intimate, involving personal issues (eg, causes of stress) and raw emotions. The way people of color cope with discrimination through social media also provides a clue. In this context, people of color see private messaging as a safe space where they can vent authentic emotions and personal experiences with family and friends, through which they receive comfort and advice, which boosts positive emotions [22]. Given that many interactions taking place through directed communication involve intimate topics and emotional support between best or close friends [23], we hypothesized that intimate directed communication should contribute to lower mental health problems by soliciting social support.

- Hypothesis 1: intimate directed communication would be associated with more social support, which, in turn, would relate to lower depression and anxiety.

In broadcasting, social support is experienced when broadcasters receive “likes,” comments, and positive feedback; these are digital indicators of empathy, acceptance, and validation [18,22]. When youths reveal intimate information to their social media network, they usually receive supportive responses from the audience, which promotes their well-being [19]. For instance, when sexual minority youths openly and authentically share their sexual identity on Instagram, they also report receiving more online peer support, which is correlated with higher levels of self-acceptance [24]. Youths with major illness, such as cancer, also take advantage of social media posting; they post pictures showing changes in appearance when they go through treatments and use the support in response to the posts to negotiate body image [25]. Teenagers also post about sensitive personal experiences on social media (eg, sexting or encounter of unwanted explicit content) as a way to seek support and advice [26]. Indeed, when broadcasters express more emotions and share intimate information, their posts become effective vehicles for relationship development and increase viewers’ intention to provide support [12,13]. Thus, intimate broadcasting has the potential to alleviate mental health problems by inducing social support.

- Hypothesis 2: intimate broadcasting would be associated with more social support, which, in turn, would relate to lower depression and anxiety.

However, intimate broadcasting comes with a risk—teenagers who perform this activity may be especially vulnerable to approval anxiety. Approval anxiety stems from concerns about online image judgment or rejection and is associated with poor psychosocial well-being [27]. This is a common anxiety associated with broadcasting in general. Teenagers today sense the need to be perfect on social media to avoid criticism, and they worry about how a “wrong” self-presentation can lead to negative evaluation [20]. The fear drives some adolescents to constantly check their profiles to ensure only the “right” things have been posted [28]. Revealing intimate information on social media can easily damage a perfect or "right" image. In fact, given the public nature of broadcasting, sharing intimate and sensitive information is not endorsed by most youths [29]. It makes intimate broadcasting nonnormative and, when considered inappropriate, can lead to negative judgments [13,30]. Teenagers who engage in intimate broadcasting may be aware of the norm and thus become especially concerned about how their posts would be appraised.

- Hypothesis 3: intimate broadcasting would be associated with stronger approval anxiety, which, in turn, would relate to greater depression and anxiety.

It is well-documented that positive broadcasting enhances broadcasters’ self-esteem [31,32], which improves one’s mental health [33]. This exercise may also contribute to better mental health through social mediators, such as social support, but this possibility has not been carefully examined. Not attending to this potential social mediator appears to be a missed opportunity, because research has shown that teenagers appreciate how social media allows them to easily reach a large audience, including friends who are both near and far, which increases their perceived online support [34]. But how might positive broadcasting relate to social support? It has been observed that teenagers’ social media responses to friends’ posts are characterized by excessive adoration and passionate appreciation [35]. It is reasonable to hypothesize that the more one broadcasts positive posts, the more such compliments they will receive, leading to a greater sense of support. Indeed, positive online self-presentation has been shown to be associated with more positive feedback from peers [19]. Similarly, when teenagers share body-positive posts featuring body acceptance, self-care, and self-love, it is correlated with receiving positive appearance comments [36]. Thus, we proposed the following hypothesis:

- Hypothesis 4: positive broadcasting would be associated with more social support, which, in turn, would relate to lower depression and anxiety.

Social comparison, defined as comparing oneself with others as a way to gain self-knowledge in social settings [37], is a common process on social media [38]. When teenagers browse peers’ posts and pages characterized by high positivity, they engage in constant self-appraisal [17,28], which creates a bidirectional association with envy [39]. Because such comparisons are usually upward [38], it can result in feelings of inferiority and self-doubt, and thus diminishes well-being [5,28,40]. People who frequently engage in upward social comparison on social media also report higher stress and fear of missing out, as well as lower self-esteem and life satisfaction, which may explain why social comparison is associated with greater anxiety and depression [41]. While adults also engage in social comparison on social media, adolescents may be especially vulnerable to this process, because many social media features align with their developmental interests in peer relationships, peer status, and identity [42].

- Hypothesis 5: positive content consumption would be associated with more social comparison, which, in turn, would relate to higher depression and anxiety.

**Figure 1:**
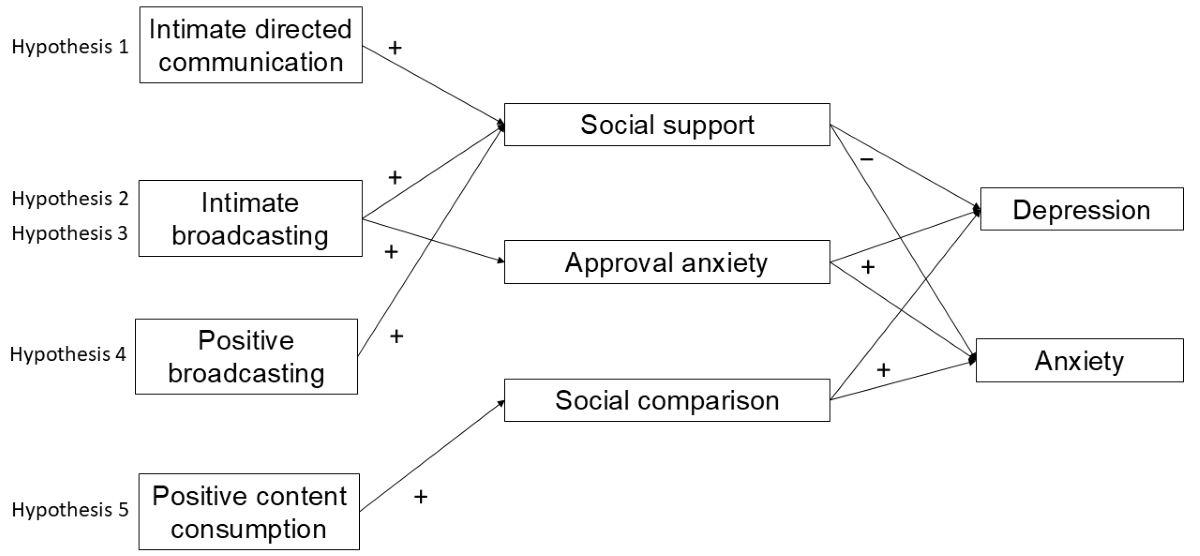
Hypothesized conceptual model.

To the best of our knowledge, this is the first study to examine 4 social media activities, with specified content characteristics, alongside 3 protective and risk processes. Results of the study should allow us to more clearly describe the opportunities and risks brought by social media. See Figure 1 for the hypothesized model.

## Methods

### Participants and Procedures

In this institutional review board (IRB)–approved study, we recruited US adolescents aged 12 to 18 years using Qualtrics’ survey panel service. Participants were required to pass at least 2 of the 3 embedded attention checks for their data to be deemed valid, and invalid responses were excluded from the analysis. Ultimately, we obtained valid data from 2105 adolescents (mean age 15.39, SD 1.82 y). The sample’s gender and racial or ethnic compositions closely reflected national distributions, with 49.35% (1039/2105) identifying as female; 63.37% (1334/2105) identifying as White or European American, 20.19% (425/2105) identifying as Latine, Hispanic, or Mexican American, 15.72% (331/2105) identifying as Black or African American; 6.13% (129/2105) identifying as Asian or Asian American; and 1.71% (36/2105) identifying as American Indian or Alaska Native. The age distribution was relatively even across age groups (14%-17%), except for those aged 12 years, who made up about 5% of the sample. Participants reported their most frequently used social media, selecting from a provided list (Facebook, Instagram, TikTok, Snapchat, and X [formerly known as Twitter]) or adding other options as needed. They could select more than one. The top 3 selections were TikTok (1287/2105, 61.14% of the sample), Instagram (816/2105, 38.76%), and Snapchat (738/2105, 35.06%). Participants also indicated their average daily social media use via a pull-down menu with 1-hour increments (1=0 to 1 hour; 13=more than 12 hours). The average reported usage was 4.2 (SD 2.78) hours (corresponding to 3-4 hours), with the most common durations being 1 to 2 hours (418/2105, 19.86%) and 2 to 3 hours (381/2105, 18.1%) per day.

### Measures

#### Overview

Measures consisted of 9 instruments: 4 for social media activities, 3 for mediators, and 2 for mental health outcomes (Cronbach α=0.83-0.91). We provide a brief description of each scale subsequently. Details of the 9 measures, including all scale items, can be found in Table 1, with descriptive statistics and correlations provided in Table 2.

**Table 1:** Results of confirmatory factor analysis^a^.

Scale and item			Factor loadings		Cronbach α
**Intimate directed communication: “** **When I message/text my friends, I...”**					0.88
	Openly share my feelings	0.81			
	Openly share things about myself	0.86			
	Openly share my thoughts	0.77			
	Feel comfortable showing my weaknesses	0.69			
	Feel comfortable talking about personal things	0.72			
**Intimate broadcasting: “** **When I post on social media, I...”**					0.88
	Openly share my feelings	0.80			
	Openly share things about myself	0.84			
	Openly share my thoughts	0.78			
	Feel comfortable showing my weaknesses	0.69			
	Feel comfortable talking about personal things	0.73			
**Positive broadcasting: “In general, how well do the following statements describe your social media posts?”**					0.89
	I look nice in my social media posts	0.67			
	My social media posts show that I have a lot of fun	0.83			
	My social media posts show my achievements	0.72			
	I post about the fun things I do with friends	0.85			
	I post on social media about the happy moments I have	0.84			
**Positive content consumption: “In general, how much do you agree that the following statements reflect what you see on social media?”**					0.88
	People post attractive pictures or videos of themselves on social media	0.74			
	People post things they are proud of on social media	0.81			
	People post good things that have happened to them on social media	0.80			
	People post things that make them look good on social media	0.80			
	If you look at people’s social media posts, it’s easy to believe they have a lot of fun	0.67			
	If you look at people’s social media posts, it’s easy to believe they have a great life	0.57			
**Social support**					0.83
	I can find help on social media	0.61			
	I can find the emotional help and support that I need on social media	0.67			
	I can talk with someone on social media about my problems	0.80			
	I can find someone on social media that helps me make decisions	0.77			
**Approval anxiety**					0.91
	I am nervous about how people will respond to my posts and photos	0.86			
	I feel anxious about how others will respond when I share a new photo on social media	0.91			
	I feel nervous after I share a new post or photo to see how others responded to it	0.88			
	I feel nervous about how others will respond when I post new updates on social media	0.85			
	I put a lot of effort into finding or creating a photo that others will approve of when I post it online	0.61			
	I put a lot of effort into composing messages and posts I share online	0.55			
**Social comparison**					0.83
	When using social media, I compare how my loved ones (romantic partner, family members, etc.) are doing with how others are doing	0.61			
	When using social media, I compare how I do things with how others do things	0.82			
	On social media, I compare what I have done with others as a way to find out how well I have done something	0.78			
	On social media, I compare how I am doing socially with other people	0.74			
**Depression**					0.84
	My appetite is poor	0.51			
	I have trouble keeping my mind on what I am doing	0.59			
	I feel depressed	0.83			
	I feel that everything I do is an effort	0.46			
	My sleep is restless	0.59			
	I feel sad	0.84			
	I cannot get “going”	0.74			
**Anxiety**					0.91
	Feeling nervous, anxious, or on edge	0.84			
	Not being able to stop or control worrying	0.87			
	Worrying too much about different things	0.86			
	Trouble relaxing	0.77			
	Being so restless that it is hard to sit still	0.71			
	Becoming easily annoyed or irritable	0.65			
	Feeling afraid as if something awful might happen	0.73			

^a^Intimate directed communication, intimate broadcasting, positive broadcasting, and positive content consumption collectively form the Content-Specific Social Media Activities Scale.

**Table 2:** Descriptive statistics and correlations.

Variables		1	2	3	4	5	6	7	8	9
**1. Intimate directed communication^a^**										
	*r*	1	0.51^b^	0.35^b^	0.23^b^	0.28^b^	0.15^b^	0.23^b^	0.10^b^	0.11^b^
	*P* value	—^c^	<.001	<.001	<.001	<.001	<.001	<.001	<.001	<.001
**2. Intimate broadcasting^d^**										
	*R*	0.51^b^	1	0.43^b^	0.07^e^	0.32^b^	0.16^b^	0.18^b^	0.05^f^	0.09^b^
	*P* value	<.001	—	<.001	.002	<.001	<.001	<.001	.03	<.001
**3. Positive broadcasting^g^**										
	*R*	0.35^b^	0.43^b^	1	0.32^b^	0.28^b^	0.26^b^	0.26^b^	–0.02	0.04
	*P* value	<.001	<.001	—	<.001	<.001	<.001	<.001	.30	.06
**4. Positive content consumption^h^**										
	*r*	0.23^b^	0.07^e^	0.32^b^	1	0.17^b^	0.18^b^	0.26^b^	0.13^b^	0.06^e^
	*P* value	<.001	.002	<.001	—	<.001	<.001	<.001	<.001	.005
**5. Social support^i^**										
	*r*	0.28^b^	0.32^b^	0.28^b^	0.17^b^	1	0.19^b^	0.27^b^	0.03	0.06^e^
	*P* value	<.001	<.001	<.001	<.001	—	<.001	<.001	.13	.005
**6. Approval anxiety^j^**										
	*r*	0.15^b^	0.16^b^	0.26^b^	0.18^b^	0.19^b^	1	0.52^b^	0.34^b^	0.38^b^
	*P* value	<.001	<.001	<.001	<.001	<.001	—	<.001	<.001	<.001
**7. Social comparison^k^**										
	*R*	0.23^b^	0.18^b^	0.26^b^	0.26^b^	0.27^b^	0.52^b^	1	0.31^b^	0.32^b^
	*P* value	<.001	<.001	<.001	<.001	<.001	<.001	—	<.001	<.001
**8. Depression^l^**										
	*r*	0.10^b^	0.05^f^	–0.02	0.13^b^	0.03	0.34^b^	0.31^b^	1	0.71^b^
	*P* value	<.001	.03	.30	<.001	.13	<.001	<.001	—	<.001
**9. Anxiety^m^**										
	*r*	0.11^b^	0.09^b^	0.04	0.06^e^	0.06^e^	0.38^b^	0.32^b^	0.71^b^	1
	*P* value	<.001	<.001	.06	.005	.005	<.001	<.001	<.001	—

^a^Mean 2.59 (SD 1.04); scale length: 5 pt.

^b^*P*<.001.

^c^Not applicable.

^d^Mean 1.86 (SD 0.90); scale length: 5 pt.

^e^*P*<.01.

^f^*P*<.05.

^g^Mean 2.89 (SD 1.14); scale length: 5 pt.

^h^Mean 4.06 (SD 0.89); scale length: 5 pt.

^i^Mean 3.02 (SD 1.08); scale length: 5 pt.

^j^Mean 2.82 (SD 1.11); scale length: 5 pt.

^k^Mean 2.76 (SD 1.03); scale length: 5 pt.

^l^Mean 2.42 (SD 0.68); scale length: 4 pt.

^m^Mean 1.19 (SD 0.89); scale length: 4 pt.

#### Social Media Activities

The 4 social media activities were assessed using 5 to 6 items each from the Content-Specific Social Media Activities Scale, developed by our research team. Scale development was informed by existing instruments measuring youths’ social media use [11,43]. Participants reported frequency of engaging in each described activity (1=never; 5=a lot). Higher average scores indicated more frequent engagement.

#### Social Support

Social support was measured using the 4-item Facebook Social Support Scale [14]. The original scale assessed participants’ agreement with statements about receiving social support on Facebook when feeling down or facing a difficult situation (1=strongly disagree; 5=strongly agree). To broaden applicability, we replaced “Facebook” with “social media.” Higher average scores indicated greater social support received through social media.

#### Approval Anxiety

Approval anxiety was measured using the 6-item Approval Anxiety Subscale of the Multidimensional Digital Stress Scale [44]. Participants were instructed to report the frequency of experiencing approval anxiety over the past 7 days (1=never; 5=always). Higher average scores indicated greater approval anxiety.

#### Social Comparison

Social comparison was measured using the 5-item Social Media Social Comparison Scale—Ability [45]. The scale measures the extent to which people compare themselves with others when using social media (1=not applicable at all, 5=extremely applicable). Higher average scores indicated greater engagement in social comparison.

#### Depression

Depression was measured using the 7-item Center for Epidemiologic Studies Depression Scale short form [46]. Participants indicated how they had been feeling recently (1=strongly disagree; 4=strongly agree). Higher average scores indicated more depressive symptoms.

#### Anxiety

Anxiety was measured using the 7-item Generalized Anxiety Disorder scale [47]. Participants reported how often they had been bothered by the described problems over the past 2 weeks (1=not at all, 4=nearly every day). Higher average scores indicated higher levels of anxiety.

### Plan for Analysis

We performed a 2-step structural equation modeling, using Mplus (version 8; Muthén & Muthén), with MLR being the estimator. We started with a confirmatory factor analysis on the 9 instruments, followed by a path analysis where the relationships among the 9 factors were modeled.

While we had proposed 5 hypotheses grounded in the MMSMU and earlier research, we recognized that some mediated paths not discussed in the MMSMU may also exist. For example, when teenagers review peers’ highlight reels, the sense of inadequacy may prompt them to wonder whether their own posts are “good enough.” In other words, positive content consumption may also trigger approval anxiety. To not miss these associations, we took an exploratory approach in our path analysis, modeling paths between all 4 social media activities and all 3 mediators. To control for the potentially inflated type 1 error rate because of multiple comparisons, we adopted the false discovery rate approach, setting the familywise type 1 error rate at 0.05. This approach compared the adjusted type 1 error rate with the *P* value to determine the significance of each path [48]. Following this plan, we regressed depression and anxiety on the 3 mediators and the 4 social media measures. All the mediators were also regressed on the 4 social media activities. The error terms of the mediators were allowed to correlate and so were the error terms of the mental health outcomes. The effects of gender, age, race or ethnicity, and amount of phone use on depression and anxiety were controlled.

Model fit was deemed acceptable when the following criteria were met: the comparative fit index (CFI) and Tucker-Lewis index (TLI) values were close to or above 0.95, and the root mean square error of approximation (RMSEA) value were lesser than 0.08. Factor loadings in the confirmatory factor analysis were expected to be 0.45 or higher.

### Ethical Considerations

The study was approved by the first author’s IRB of the Social, Behavior, and Educational Committee (IRB-23-76). Parental consent and adolescent assent were obtained before participants could access the survey. Participants were compensated according to preestablished agreements with their panel provider.

## Results

### Confirmatory Factor Analysis

One reversed item assessing social comparison was excluded because of low factor loading (0.14). Following the modification indexes, we correlated one pair of item within each of the following factors: positive content consumption, social support, and approval anxiety. After these modifications, the model fit well and confirmed the presumed structure: χ^2^_1088_=3600.1; *P*<.001; RMSEA=0.033, 90% CI 0.032-0.034; CFI=0.949; TLI=0.945. See Table 1 for factor loadings and Cronbach α values and Table 2 for descriptive statistics and correlations.

### Path Analysis

**Figure 2:**
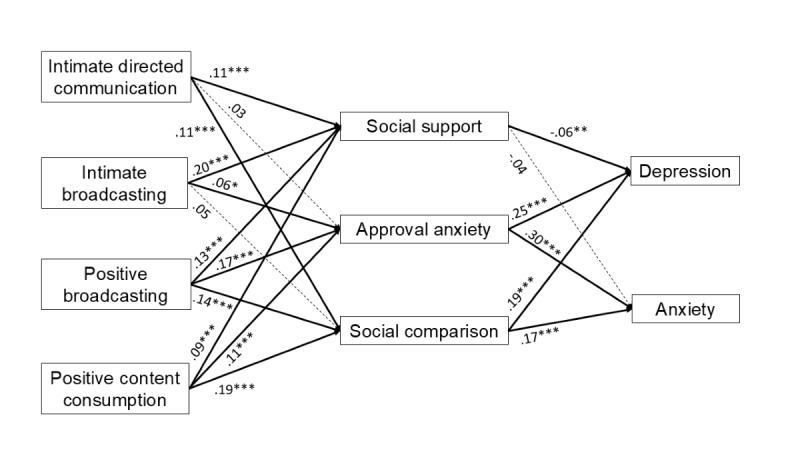
Standardized path coefficients between social media activities, mechanisms, and well-being outcomes. The solid bolded line denotes a significant indirect path. The dashed line denotes a nonsignificant path. For clarity, controlled paths and the direct paths from social media activities to mental health outcomes are not displayed. *P<.05, **P<.01, ***P<.001.

The proposed model showed good fit after we added the path from gender to approval anxiety: χ^2^_11_=38.1; *P*<.001; RMSEA=0.034, 90% CI 0.023-0.046; CFI=0.991; TLI=0.961. The direct associations between social media activities and the mediators, as well as between the mediators and mental health outcomes, are as follows (Figure 2). Intimate directed communication had a positive relationship with social support (β=.11; *P*<.001) and social comparison (β=.11; *P*<.001), but it was not associated with approval anxiety (β=.03; *P*=.27). Intimate broadcasting was associated with higher social support (β=.20; *P*<.001) and approval anxiety (β=.06; *P*=.02) but not social comparison (β=.05; *P*=.045, using the false discovery rate approach, the *P* value of this path had to be under .04 to be significant). Positive broadcasting was related to greater social support (β=.13; *P*<.001), approval anxiety (β=.17; *P*<.001), and social comparison (β=.14; *P*<.001). Positive content consumption was also associated with greater social support (β=.09; *P*<.001), approval anxiety (β=.11; *P*<.001), and social comparison (β=.19; *P*<.001). Social support was associated with lower depression (β=–.06; *P*=.007) but not anxiety (β=–.04; *P*=.06). Approval anxiety (depression: β=.25; *P*<.001; anxiety: β=.30; *P*<.001) and social comparison (depression: β=.19; *P*<.001; anxiety: β=.17; *P*<.001) were both related to higher depression and anxiety.

Of particular interest were the direct, indirect, and total paths from the social media variables to the mental health outcomes (Table 3). Intimate directed communication was not directly associated with depression (β=.03; *P*=.17) or anxiety (β=.03; *P*=.20). It was indirectly associated with lower depression (but not anxiety) through social support (β=–.01; *P*=.02). It also had an indirect relationship with greater depression (β=.02; *P*<.001) and anxiety (β=.02; *P*<.001) via social comparison. Neither indirect paths through approval anxiety were significant. Collectively, the total paths from intimate directed communication to depression (β=.06; *P*=.03) and anxiety (β=.06; *P*=.04) were both significant.

Intimate broadcasting was not directly related to depression (β=.04; *P*=.12) or anxiety (β=.05; *P*=.06). It was indirectly associated with lower depression (but not anxiety) via social support (β=–.01; *P*=.01). It was also indirectly related to greater depression (β=.02; *P*=.02) and anxiety (β=.02; *P*=.02) through approval anxiety. Neither indirect paths through social comparison were significant. For the total paths, only the one to anxiety was significant (β=.07; *P*=.02).

Positive broadcasting had a direct association with lower depression (β=–.21; *P*<.001) and anxiety (β=–.13; *P*<.001). It had an indirect association with lower depression (but not anxiety) through social support (β=–.01; *P*=.02), but it was also indirectly related to greater mental health problems through approval anxiety (depression: β=.04; *P*<.001; anxiety: β=.05; *P*<.001) and social comparison (depression: β=.03; *P*<.001; anxiety: β=.03; *P*<.001). Collectively, the total paths from positive broadcasting to lower depression (β=–.14; *P*<.001) and anxiety (β=–.06; *P*=.03) were both significant.

Positive content consumption had a direct association with higher depression (β=.07; *P*=.001) but not anxiety (β=–.03; *P*=.25). It had an indirect association with lower depression (but not anxiety) through social support (β=–.01; *P*=.02), but it was also indirectly related to greater mental health problems through approval anxiety (depression: β=.03; *P*<.001; anxiety: β=.03; *P*<.001) and social comparison (depression: β=.04; *P*<.001; anxiety: β=.03; *P*<.001). Collectively, the total paths from positive content consumption to higher depression (but not anxiety) was significant (β=.13; *P*<.001).

Several controlled paths were significant. Female participants reported higher approval anxiety (β=.20; *P*<.001), depression (β=.19; *P*<.001), and anxiety (β=.21; *P*<.001). Older teenagers reported greater depression (β=.04; *P*=.04). Race and ethnicity (identifying as White; depression: β=.13; *P*=.002; anxiety: β=.11; *P*=.007) and amount of phone use (depression: β=.15; *P*<.001; anxiety: β=.12; *P*<.001) were related to greater depression and anxiety.

**Table 3:** Direct, indirect, and total paths from social media activities to depression and anxiety.

Path	Standardized coefficients, β				
	Direct path	Indirect path through social support	Indirect path through approval anxiety	Indirect path through social comparison	Total path
Intimate directed communication → depression	.03	–.01^a^	.01	.02^b^	.06^a^
Intimate directed communication → anxiety	.03	–.01	.01	.02^b^	.06^a^
Intimate broadcasting → depression	.04	–.01^a^	.02^a^	.01	.05
Intimate broadcasting → anxiety	.05	–.01	.02^a^	.01	.07^a^
Positive broadcasting → depression	–.21^b^	–.01^a^	.04^b^	.03^b^	–.14^b^
Positive broadcasting → anxiety	–.13^b^	–.01	.05^b^	.03^b^	–.06^a^
Positive content consumption → depression	.07^c^	–.01^a^	.03^b^	.04^b^	.13^b^
Positive content consumption → anxiety	–.03	–.00	.03^b^	.03^b^	.04

^a^*P*<.05.

^b^*P*<.001.

^c^*P*<.01.

## Discussion

Overall, intimate directed communication, intimate broadcasting, and positive content consumption were all associated with greater mental health problems through approval anxiety or social comparison. Positive broadcasting, on the other hand, was associated with lower mental health concerns, mostly due to its direct association with lower depression and anxiety.

### Intimate Directed Communication

Intimate directed communication was associated with greater depression and anxiety through its activation of social comparison. Teenagers’ intimate directed communication may revolve around developmentally relevant themes, such as family and friends, self-concept and self-evaluation, emotions, sexual activities, and even antisocial behaviors [49,50]. Given the salience of identity development during adolescence [51] and the crucial role of social comparison in gaining self-knowledge and informing identity [37,52], social comparison may be an unplanned result when teenagers engage in these digital conversations with peers. Although adolescents may initiate intimate directed communication to seek support and feedback rather than for social comparison, some research suggests that feedback seeking and social comparison are interconnected processes [53]. In addition, even without the intention to engage in social comparison, the process can occur spontaneously [52]. This is a route that has been largely overlooked in existing literature.

Although social support acted as a protective factor against depression, its coefficient was too small to counterbalance or nullify the negative paths. In fact, this pattern was observed across all 4 social media activities. A possible reason is that we focused on social support available on social media, whereas a more comprehensive scale measuring both online and offline social support would have been more ideal. Indeed, a few studies taking this comprehensive approach have shown that social support is a robust mediator between social media use and various well-being outcomes [22,54]. We chose the current scale for its good reliability and brevity, the latter of which was important to prevent exhausting the adolescent participants. We urge readers to not interpret our findings as evidence that social support is unimportant, as it may play a significant role in other contexts.

### Intimate Broadcasting

Intimate broadcasting was associated with more mental health problems (especially anxiety) via approval anxiety. Approval anxiety is a growing concern for many adolescents [28], especially when they decide to broadcast information on social media (as opposed to sharing it through private messaging) [20]. When adolescents engage in intimate broadcasting, they are open about themselves, sharing personal, private, or sensitive self-information with a large audience. While such self-presentation can be deemed authentic and solicits support from the social media audience [12,24,25], it can also be viewed as inappropriate and against social norms (eg, oversharing). In the latter case, intimate broadcasting makes the audience feel less connected to the broadcaster and reduces the broadcaster’s credibility and social attractiveness [13,30]. The broadcasters may be aware of the risk and thus feel especially anxious about rejection. Furthermore, when teenagers engage in intimate broadcasting, they might share aspects core to their sense of self, such as sexual orientation, challenging experiences in important relationships, or major illness [24,25]. Given that supportive social media comments indicate validation [18,22], negative comments or the absence of supportive feedback in these cases can feel like a rejection of teenagers’ identity. It is thus not surprising that teenagers who engaged in more intimate broadcasting were more anxious about the judgment they would receive. Unfortunately, if teenagers receive negative comments, those remarks linger in their thoughts for a long time, and lack of affirming feedback can also cause negative emotions [17]. The persistent thoughts of criticism and negative emotions may fuel future approval anxiety.

### Positive Broadcasting

Positive broadcasting highlights teenagers’ uplifting experiences, achievements, and friendships. Interestingly, it was related to fewer mental health problems primarily through the direct paths rather than social support. This is an example of social media self-effects independent of social feedback [55,56]. The direct path likely holds because positive broadcasting affirms one’s identity. As youths broadcast what they enjoy or are proud of, their attention is selectively focused on these positive events or traits. This biased scanning helps people maintain a positive self-image and promotes well-being, especially in the face of stress and challenges [56,57]. Such practice also helps broadcasters recognize and enjoy positive moments in their everyday lives, which enhances their appreciation for life and, in turn, predicts greater happiness [58]. Although our findings are correlational rather than causal, and thus we cannot rule out the possibility that teenagers with better mental health tend to have more positive materials to broadcast, there have been experimental studies demonstrating that positive self-presentation does improve well-being [31,32,58], and there has been longitudinal research showing the reciprocal relationship between the two [24].

Somewhat surprisingly, positive broadcasting was also associated with more mental health problems through approval anxiety and social comparison. Due to the public nature of broadcasting, perhaps it is inevitable for teenagers to consider the audience’s reactions (approval anxiety) and compare their achievements and experiences with others’ (social comparison). In fact, some young people, such as those who are less mindful, may attach great importance to positive broadcasting. They might question their own worth and peer acceptance if their carefully curated image is not accepted and validated by their audience [11]. Despite this drawback, the overall benefits of positive broadcasting still outweighed the risks, and this activity helped protect teenagers from depression and anxiety.

### Positive Content Consumption

Positive content consumption, or being exposed to other users’ highlight reels, had the highest mean score among all social media variables. It resonates with the well-documented positivity bias on social media [17,18]. This activity was related to more mental health problems, particularly depression, both directly and indirectly via approval anxiety and social comparison. Both the direct association between positive content consumption and poor mental health [59] as well as the indirect path through social comparison [5,40] have been identified in earlier research. It is noteworthy that the act of social comparison and negative feelings stemming from it appear to have a cyclical relationship. For instance, envious feelings not only result from social comparison but also prompt individuals to engage in more social comparison while browsing social media [39].

While the role of social comparison has been well recognized in social media research, approval anxiety is rarely discussed as a factor mediating the relationship between browsing and poor mental health. However, our results showed that the mediating effect sizes of the two were comparable. It suggests that when teenagers view their peers’ celebrated moments, they not only compare themselves to others but also worry about not meeting their peers’ standards. Observing the number of likes and followers their peers have may heighten this anxiety, leading teenagers to worry about being judged if they do not measure up. Interestingly, research indicates that those who engage in frequent upward social comparison also spend more time censoring social media platforms to avoid judgment [41]. It suggests that social comparison and approval anxiety may be 2 processes operating in tandem, and thus the social media activities that trigger social comparison could also easily trigger approval anxiety.

It was surprising that positive content consumption contributed to less depression by boosting social support. Scholars have argued that communication devices reflect one’s relational networks, and the mere presence of the devices can evoke awareness of these connections [60]. Social media can reduce loneliness, regardless of whether users receive feedback, as it brings to mind their existing social bonds [55]. Similarly, content consumption may serve this function; by viewing friends’ posts, teenagers become aware of the support available to them if needed. Another explanation is that the positive content may be encountered in identity-based social media groups (eg, sports team pages and racial and ethnic communities). Just as members of such groups can experience vicarious discrimination when witnessing fellow members facing it [61], they may also sense vicarious support when they see positive events from others in their communities. Overall, however, the risks of positive content consumption still outweighed the small benefits, rendering it an activity detrimental to adolescent mental health.

### Limitations

We have noticed several limitations to our study. First, this is a cross-sectional study; therefore, the causal relationship among variables cannot be determined. Although we interpreted our findings within our theoretical framework—suggesting that specific social media activities influence mental health through protective and risk mechanisms—we cannot rule out the possibility that adolescents with varying levels of mental health problems engage with social media in systematically different ways. Longitudinal and experimental studies have shown that poor psychosocial states, such as social isolation, social anxiety, and depression, can predict specific patterns of technology use, including disordered internet use and willingness to perform intimate broadcasting [62,63]. In addition, depressed teenagers are more preoccupied with social media feedback, feel more rejected in online communication, and feel more insecure after scrolling [64]. These findings suggest that mental health and well-being status may drive social media behaviors and shape digital experiences. Thus, an alternative interpretation of our results is that adolescents experiencing greater mental health challenges may be more prone to approval anxiety and social comparison, leading them to engage more in intimate directed communication, intimate broadcasting, and positive content consumption. Conversely, those with fewer mental health concerns may have more positive experiences to share, resulting in greater engagement in positive broadcasting (but see our earlier discussion, where experimental evidence supporting our hypothesized directionality is provided). We recommend continued longitudinal and experimental research to further clarify the directionality of the path model.

Second, to keep the model parsimonious, we included a limited number of 4 social media activities. We chose these activities given their prevalence among and developmental implications for youth. However, future research may consider including additional activities. For instance, negative broadcasting, such as hate speech, may do harm to mental health by decreasing perceived social support. A comparison between positive broadcasting and negative broadcasting would provide scholars additional insight. Third, as mentioned earlier, our exclusive focus on online social support may have underestimated the benefits of social support. To uncover the full impact of social support, we recommend using scales assessing different types of support from different sources, both online and offline [65]. Such scales would also allow scholars to differentiate between the impacts of various types or sources of support, should that be of interest. Finally, when interpreting our results, we drew on many studies where adolescents’ main interactants or audience members are those with whom they have some connections (eg, friends and schoolmates). However, adolescents’ social media networks may extend beyond these familiar circles to include hobby groups, social communities, and influencers. While we noted how following an identity-based group might facilitate vicarious support in positive content consumption, our study did not directly account for this broader network composition. The makeup of one’s network likely influences the content encountered and its perceived personal relevance, thereby shaping its emotional impact [66]. Future research should examine how social media network composition shapes one’s digital experiences.

### Implications and Contributions

Despite the limitations, the study offers significant theoretical and practical contributions. At the theoretical level, the study expands the research focus from merely the amount or dichotomy of social media use to a nuanced examination of 4 distinct activities. Importantly, it considers the content characteristics associated with these engagements. By doing so, the current research responds to the call for more attention to the content of youths’ social media use [3,7,8] and expands the MMSMU [9]. This approach provides better conceptual clarity and allows for a more nuanced analysis of how different types of social media practices are related to user experiences and well-being.

Furthermore, the study clarifies the mechanisms through which the 4 social media usages associate with mental health problems. In addition to the expected mediating paths (ie, the ones introduced in our hypotheses), we identified a few understudied paths. These include the ones from intimate directed communication to social comparison, positive broadcasting to approval anxiety and social comparison, and positive content consumption to approval anxiety. The implications are 3-fold. First, the findings suggest that directed communication and broadcasting, which are usually grouped as “active” social media use are distinctive [5]. Although we studied intimate practices for both, directed communication took the indirect route through social comparison, whereas broadcasting contributed to poor mental health through approval anxiety. Second, approval anxiety merits further investigation. Among the 4 social media activities, 3 activities were associated with poor mental health through approval anxiety. Quite a few scholars have offered valuable insights into what this anxiety feels like [17,20,27]. The next step may be to investigate the predictors of this digital stress. For example, given teenagers’ desires for peer acceptance and status, adolescence may be a developmental period with a heightened risk for approval [9]. In addition, since negative comments resonate in one’s thoughts longer than positive ones [17], individuals who receive more negative feedback on their earlier posts may be particularly vulnerable to this anxiety. Identifying possible predictors can help inform strategies to mitigate approval anxiety and promote healthier social media interactions among adolescents. Finally, although social comparison is primarily brought up in the context of social media browsing in existing literature [5,38], our results showed that the process was relevant to directed communication and broadcasting as well. Social comparison appears to be a psychological process relevant to multiple, if not most, social media activities. However, the magnitude of its impact varies as a function of the nature of a social media activity (eg, positive content consumption had the largest direct-path coefficient) and whether the social media activity simultaneously activates a protective mechanism (such as identity affirmation, as may be the case for positive broadcasting). This highlights the need for researchers and practitioners to consider the broader social media context in which social comparison occurs and identify possible moderators that could shape its effects.

At the practical level, social media can benefit adolescents, provided they consider the mental health risks posed by their engagement. When using social media, teenagers should avoid socially comparing themselves with others, especially while engaging in positive content consumption and intimate directed communication. Instead, these activities should be used intentionally to elicit social support through interactions with both content and people. Broadcasting on social media has advantages, but teenagers should be thoughtful about the information shared on these platforms. Openly sharing personal details, thoughts, and feelings may be tempting because it fosters relational closeness and acquires social support [12,13,25]. However, adolescents should be aware that revealing intimate vulnerabilities to followers can trigger anxiety due to fear of judgment. Interestingly, our results showed that such approval anxiety was absent from intimate directed communication, operationalized as intimate interaction through private texting and messaging, which typically takes place among close friends [23]. It appears that adolescents are most concerned about being judged when their posts can be evaluated by a large group of less close associates. Thus, being selective about their audience can reduce teenagers’ fear of being ridiculed by peers and strangers [20]. Limiting intimate broadcasting to a smaller group of trustworthy friends may allow adolescents to receive social support without excessive concern about approval anxiety. Positive broadcasting is an adaptive activity for mental health, likely due to the self-effects it generates [55,56]. Therefore, it should be fine for teenagers to post what they enjoy or feel proud of. At the same time, it is imperative that adolescents avoid becoming preoccupied with maintaining a perfect image or evoking likes and comments to prevent the harms of approval anxiety and social comparison.

Research has shown that teenagers desire guidance on social media use from adults [35]. To guide teenagers in navigating their social media use, parents are advised to have regular conversations with their children about social media, incorporating the recommendations outlined earlier to help them engage with online interactions mindfully. Schools should integrate digital literacy education into their curricula, ensuring that students not only critically evaluate online content but also learn strategies aligned with these recommendations to manage their social media experiences in a healthy way. Social media platforms, particularly those catering to children and adolescents, should prioritize self-directed and community-supported digital experiences, while minimizing manipulative design choices that encourage prolonged engagement at the expense of users’ mental health [67]. Social media metrics, such as likes and follower counts, exemplify these manipulative designs that may trigger approval anxiety and social comparison. Some platforms have begun allowing users to hide these quantifiable metrics, which is a positive step toward reducing potential harms. Collectively, these efforts by parents, schools, and platforms can contribute to a safer and more supportive digital environment for adolescents.

### Conclusions

In this study, we found that intimate directed communication was associated with more mental health problems through social comparison; intimate broadcasting was related to poor mental health (especially anxiety) via approval anxiety; and positive content consumption was a risk factor for mental health problems (especially depression) due to both approval anxiety and social comparison. In contrast, positive broadcasting contributed to better mental health because of its direct association with lower depression and anxiety. Findings of the study highlight the complexity of social media’s role in adolescent mental health, which suggests that the nature of social media practices significantly shape their implications for mental health. We hope the results provide both adults and adolescents with a clearer understanding of how to leverage social media’s benefits while mitigating potential risks to mental health.

## Abbreviations

CFI: comparative fit index
IRB: institutional review board
MMSMU: Multidimensional Model of Social Media Use
RMSEA: root mean square error of approximation
TLI: Tucker-Lewis index

## Data Availability

The datasets generated or analyzed during this study are not publicly available because the overall project is still ongoing, and the data are being used for additional manuscripts. However, they are available from the corresponding author upon reasonable request.
